# Cigarette smoke restricts the ability of mesenchymal cells to support lung epithelial organoid formation

**DOI:** 10.3389/fcell.2023.1165581

**Published:** 2023-09-19

**Authors:** P. P. S. J. Khedoe, W. A. A. M. van Schadewijk, M. Schwiening, J. P. Ng-Blichfeldt, S. J. Marciniak, J. Stolk, R. Gosens, P. S. Hiemstra

**Affiliations:** ^1^ Department of Pulmonology, Leiden University Medical Centre, Leiden, Netherlands; ^2^ Department of Medicine, Cambridge Institute for Medical Research, University of Cambridge, Cambridge, United Kingdom; ^3^ Department of Molecular Pharmacology, Groningen Research Institute of Pharmacy, University of Groningen, Groningen, Netherlands; ^4^ MRC Laboratory of Molecular Biology, Cambridge Biomedical Campus, Cambridge, United Kingdom

**Keywords:** cigarette smoke extract, lung epithelial organoid, COPD, fibroblasts, oxidative stress, ER stress

## Abstract

Adequate lung epithelial repair relies on supportive interactions within the epithelial niche, including interactions with WNT-responsive fibroblasts. In fibroblasts from patients with chronic obstructive pulmonary disease (COPD) or upon *in vitro* cigarette smoke exposure, Wnt/β-catenin signalling is distorted, which may affect interactions between epithelial cells and fibroblasts resulting in inadequate lung repair. We hypothesized that cigarette smoke (CS), the main risk factor for COPD, interferes with Wnt/β-catenin signalling in fibroblasts through induction of cellular stress responses, including oxidative- and endoplasmic reticulum (ER) stress, and thereby alters epithelial repair support potential. Therefore, we assessed the effect of CS-exposure and the ER stress inducer Thapsigargin (Tg) on Wnt/β-catenin signalling activation in MRC-5 fibroblasts, and on their ability to support lung epithelial organoid formation. Exposure of MRC-5 cells for 15 min with 5 AU/mL CS extract (CSE), and subsequent 6 h incubation induced oxidative stress (*HMOX1*). Whereas stimulation with 100 nM Tg increased markers of both the integrated stress response (ISR - *GADD34*/*PPP1R15A*, *CHOP*) and the unfolded protein response (UPR - *XBP1spl*, *GADD34/PPP1R15A*, *CHOP* and *HSPA5/BIP*), CSE only induced *GADD34*/*PPP1R15A* expression. Strikingly, although treatment of MRC-5 cells with the Wnt activator CHIR99021 upregulated the Wnt/β-catenin target gene *AXIN2,* this response was diminished upon CSE or Tg pre-exposure, which was confirmed using a Wnt-reporter. Furthermore, pre-exposure of MRC-5 cells to CSE or Tg, restricted their ability to support organoid formation upon co-culture with murine pulmonary EpCam^+^ cells in Matrigel at day 14. This restriction was alleviated by pre-treatment with CHIR99021. We conclude that exposure of MRC-5 cells to CSE increases oxidative stress, *GADD34/PPP1R15A* expression and impairs their ability to support organoid formation. This inhibitory effect may be restored by activating the Wnt/β-catenin signalling pathway.

## Introduction

Lung injury and inadequate repair processes underlie a variety of chronic lung diseases, including chronic obstructive pulmonary disease (COPD) and interstitial lung diseases (ILD) (Kneidinger et al., 2011; Rock and Konigshoff, 2012; Baarsma et al., 2017). Adequate lung epithelial repair requires interactive cross-talk within the alveolar stem cell/progenitor niche between progenitor cells and their microenvironment, including structural cells such as fibroblasts (Baarsma et al., 2017; Skronska-Wasek et al., 2018). The mesenchymal niche orchestrates alveolar cell function and repair through secretion of fibroblast growth factors (FGFs), Wnt ligands, Wnt signalling activation and other growth factors (Frank et al., 2016; Zepp et al., 2017; Khedoe et al., 2021; Penkala et al., 2021). Dysfunctional signalling of repair pathways, including the Wnt/β-catenin pathway, has been implicated in aberrant lung epithelial repair in COPD (Rock and Konigshoff, 2012; Kneidinger et al., 2011). Importantly, Wnt-responsive mesenchymal cells dictate alveolar cell proliferation and differentiation, whereas myofibroblasts impair alveolar repair (Zepp et al., 2017). The Wnt/β-catenin signalling pathway is central to the interaction between epithelial cells and surrounding mesenchymal cells during repair processes. In lung diseases such as COPD, it has been hypothesized that this crosstalk is impaired, leading to inappropriate signalling between, for example, airway epithelial cells and fibroblasts. Wnt/β-catenin signalling is thus important for proper interaction between both lung epithelial cell and fibroblasts and its distortion may lead to disturbed cross-talk. In COPD, distorted Wnt/β-catenin signalling in fibroblasts may help to explain remodelling processes that together with tissue injury are a cause of impaired lung function (Baarsma et al., 2017).

One common cellular stress response, which is activated in smokers with and without COPD, is the unfolded protein response (UPR) to endoplasmic reticulum stress (ER stress) (Malhotra et al., 2009; Gan et al., 2011; Lawson et al., 2011; Marciniak, 2019). It has been demonstrated that ER stress may interfere with various repair pathways, which is relevant for COPD and ILD, since these pathways are overactivated in diseased lung tissue (Tanjore et al., 2012; Wei et al., 2013; Dickens et al., 2019). Whereas this cellular response is activated to restore homeostasis upon cellular or micro-environmental stress, prolonged activation can lead to cell dysfunction, inappropriate differentiation and cell death (Marciniak, 2019). Three transducers mediate sensing of ER stress and initiate activation of the UPR: ATF6, IRE1 and PERK. Autophosphorylation of the kinase domain of PERK results in attenuation of protein synthesis by phosphorylation of eukaryotic initiation factor (eIF2)-α, and this mechanism also contributes to the integrated stress response (ISR). However, in addition to PERK and ER stress, further eIF2α kinases can respond to a range of other cellular stresses to trigger the ISR resulting in similar downstream events (Emanuelli et al., 2020). Not only cigarette smoke, but also various endogenous and exogenous triggers are able to activate either the UPR or the ISR (Marciniak, 2019; Emanuelli et al., 2020; van 't Wout et al., 2015). Importantly, there is evidence for prolonged activation of the UPR and ISR in (epithelial) cells in cigarette smokers and COPD patients (Malhotra et al., 2009), and this has been implicated in dysfunction of both airway epithelial cells and pulmonary fibroblasts, leading to epithelial-to-mesenchymal transition (EMT) and myofibroblast differentiation (Delbrel et al., 2019; Song et al., 2019), respectively. Mutations in various genes, including *SFPTC* and *MUC5B* may induce ER stress in lung epithelial cells, contributing to EMT and fibrosis in IPF (Burman et al., 2018; Dickens et al., 2019). In addition to causing cellular dysfunction, prolonged activation of the UPR and ISR may interfere with mRNA processing and translation of proteins, leading to impairment of signalling pathways involved in repair.

In this study, we used an *in vitro* model of lung repair in which mesenchymal cells support epithelial organoid formation. Using this model, we have recently shown that TGF-β treatment of fibroblasts impairs their ability to support organoid formation (Ng-Blichfeldt et al., 2019). In the present study, our aim was to dissect the impact of cellular stress responses (e.g., oxidative stress and ER stress) on the supportive role of the lung fibroblast for lung epithelial cell repair. Therefore, we focused on the effect of cigarette smoke extract- and chemically-induced ER stress in MRC-5 fibroblasts, including Wnt/β-catenin signalling and their ability to support epithelial progenitor function.

## Materials and methods

### Mouse epithelial cell isolation

Lung epithelial cells were isolated from C57BL6/J mice (8–14 weeks) as described before (Ng-Blichfeldt et al., 2019; Wu et al., 2019). Mice were kept under a 12/12 h light/dark cycle and had *ad libitum* access to food and water. In short, after anesthesia (40 mg/kg ketamine and 0.5 mg/kg dexdomitor, i.p.) the pulmonary vasculature of C57BL/6N or C57BL6/J mice was flushed with PBS, after which the lungs were filled with Dispase (BD, Biosciences, Oxford, United Kingdom) in low-melting agarose (Sigma Aldrich, Poole, United Kingdom) for 45 min at room temperature (RT). Lung lobes were homogenized in DMEM containing DNase1 (Applichem, Germany), after which a single-cell suspension was prepared. Subsequently, using magnetic-activated cell sorting (MACS isolation), cell suspensions underwent a negative selection for CD45 (Miltenyi Biotec, Teterow, Germany) and CD31 (Miltenyi), after which EpCAM^+^ (CD326) epithelial cells were isolated (Miltenyi) as described before (Ng-Blichfeldt et al., 2019; Wu et al., 2019). Antibodies used for experimental procedures are listed in Table 1. EpCAM^+^ cells were resuspended in DMEM with 10% fetal calf serum (FCS, Bodinco, Alkmaar, Netherlands). Experimental animal protocols were approved by the University of Groningen animal experimentation committee under CCD license AVD105002015303.

**TABLE 1:** Antibodies for experimental procedures.

Antibody	Technique	Supplier + Cat. No.
CD45	MACS sorting	Miltenyi Biotec, Germany 130-052-301
CD31	MACS sorting	Miltenyi Biotec, Germany 130-097-418
CD326	MACS sorting	Miltenyi Biotec, Germany 130-091-051
Pro-Surfactant protein-C	Immunofluorescence staining	Millipore AB3786
Acetylated tubulin	Immunofluorescence staining	Sigma T7451
Donkey anti-Mouse Secondary Antibody, Alexa Fluor™ 568	Immunofluorescence staining	Invitrogen/Life Technologies A10037
Goat anti-Rabbit Secondary Antibody, Alexa Fluor™ 488	Immunofluorescence staining	Invitrogen/Life Technologies A11008

### MRC-5 lung fibroblast and MEF culture and stimulation

MRC-5 lung fibroblasts (ATCC CCL 171, United States) were cultured in MEM (Gibco) containing 100 U/ml penicillin/streptomycin (Gibco), 25 mM HEPES (Invitrogen), non-essential amino acids (NEAAS, Gibco) and 10% heat-inactivated FCS at 37°C in 5% CO_2_. For mRNA analyses, MRC-5 lung fibroblasts were grown to confluence and kept in serum-free medium overnight. MRC-5 were then stimulated for 15 min with 2 or 5 AU/mL freshly prepared cigarette smoke extract (CSE) generated as described in (23), washed with PBS and then further cultured for 6 h. To induce ER stress, MRC-5 fibroblasts were incubated for 6 h with 100 nM thapsigargin (Tg, Sigma). To examine the effect of Wnt/β-catenin activation on cellular stress responses (e.g., oxidative stress and ER stress), MRC-5 cells were treated with vehicle, 2 µM CHIR99021 or 10 mM N-acetylcysteine (NAC) during the 6 h exposure. After 6 h, MRC-5 cells were lysed for RNA isolation and gene expression analyses. Similar stimulation procedures were applied to mouse embryonic fibroblasts (MEFs), derived from WT, Gadd34^−/−^, Chop^−/−^ or EIF2a AA signalling deficient mice (Scheuner et al., 2001).

**FIGURE 1:**
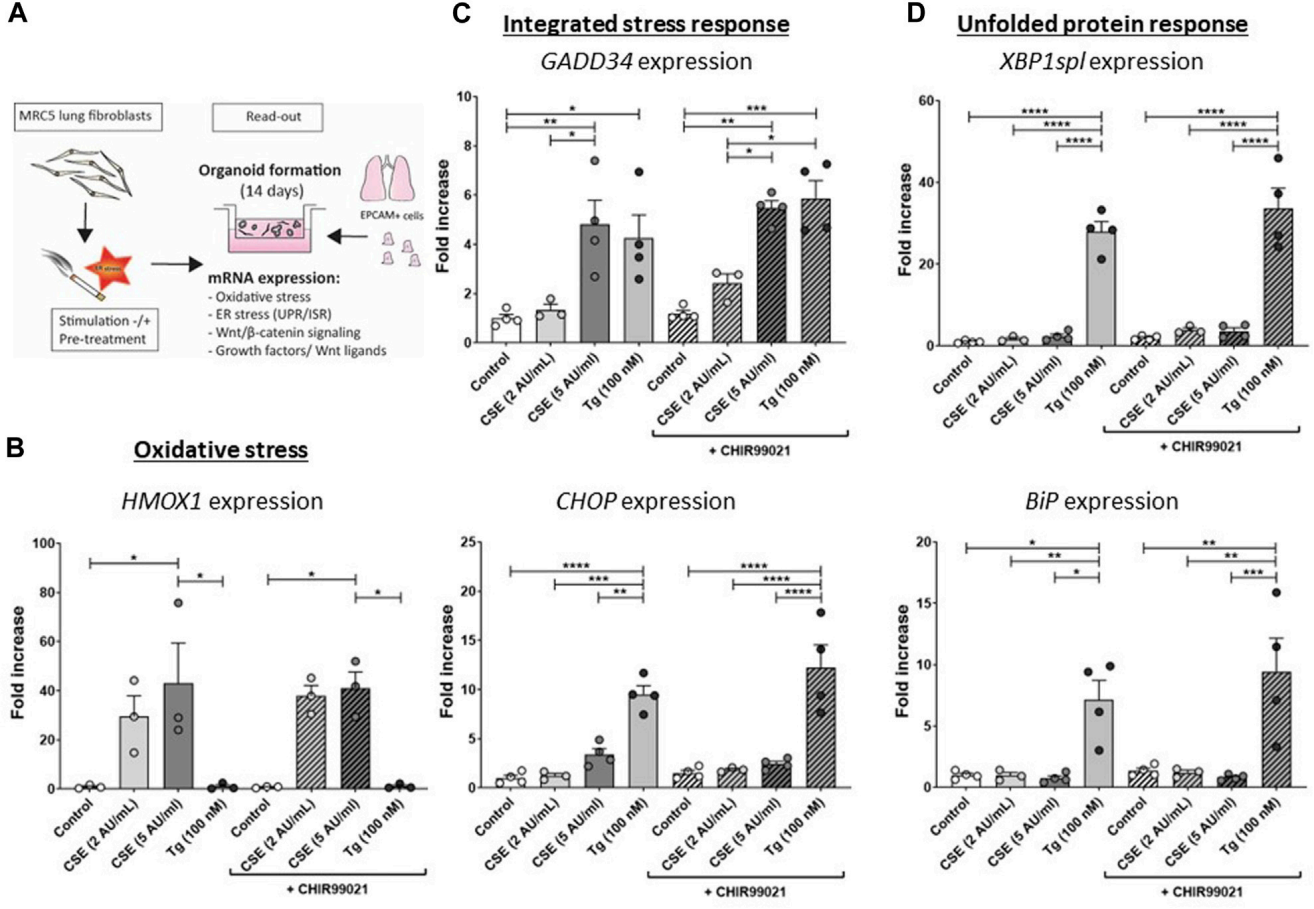
**CSE induces stress responses in MRC-5 lung fibroblasts** MRC-5 fibroblasts were cultured until confluency was reached, after which they were serum-starved for 24 h. Upon starvation, MRC-5 fibroblasts were exposed to vehicle (white dots/bars) freshly-prepared CSE (2 or 5 AU/mL, light and dark grey dots/bars resp.) for 15 min or Tg (dark grey dots/grey bars) and stimulated for 6 h. CHIR99021 or vehicle (2 μM, stripes bars) was added during stimulation with CSE or Tg (experimental set-up shown in **(A)**). Cells were then lysed for RNA isolation, cDNA synthesis and RT-qPCR. HMOX1 expression **(B)** was measured as marker for oxidative stress. GADD34 **(C)** and CHOP mRNA expression were determined as a read-out for activation of the ISR, whereas XBP1spl and BiP **(D)** were measured to determine UPR activation. Data are shown as mean ± SEM (with individual datapoints); N = 3–4 independent experiments; *p < 0.05, **p < 0.01, ***p < 0.001, ****p < 0.0001 Data were analyzed using One-way ANOVA statistical testing in Graphpad Prism 9.3.1. Abbreviations: AU/mL: arbitrary units/mL; CSE: cigarette smoke extract; ER stress: endoplasmic reticulum stress; ISR: integrated stress response; Tg: thapsigargin; UPR: unfolded protein response.

For organoid assays, MRC-5 fibroblasts were first mitotically inactivated using 10 μg/mL Mitomycin-C (Sigma-Aldrich) (Ng-Blichfeldt et al., 2019), followed by washing and a restoration period of 1 h. MRC-5 fibroblasts were then stimulated for 15 min with 5 AU/mL freshly prepared CSE (Luppi et al., 2005), washed with PBS and then incubated for 6 h, either in the presence or absence of 2 µM CHIR99021 or 10 mM NAC. ER stress was induced using 100 nM Tg for 6 h, either or not in presence of CHIR99021 or NAC. MRC-5 fibroblasts were trypsinized thereafter and immediately used for organoid assays. An overview of the experimental set-up is shown in Figure 1A.

### Organoid assay

Organoid assays were performed as previously described (Ng-Blichfeldt et al., 2019). Pre-treated or pre-exposed MRC-5 fibroblasts were resuspended in a 1:1 ratio (20.000 cells each) with EpCAM^+^ cells in 50 µL DMEM/10% FBS +50 µL growth-factor reduced Matrigel (Corning). Cell suspensions were seeded on 24-transwell inserts and subsequently incubated at 37°C for ∼30 min to enable polymerization of the Matrigel. Organoid cultures were maintained in DMEM/F12 with 5% (vol/vol) FBS, 2 mM L-glutamine, p/s, 1x insulin-transferrin-selenium (Gibco), recombinant mouse EGF (0.025 μg/mL, Sigma), bovine pituitary extract (30 μg/mL, Sigma), and freshly added all-trans retinoic acid (0.01 μM, Sigma) at 37°C with 5% CO_2_. Y-27632 (10 μM, Tocris, Oxford, United Kingdom) was added for the first 48 h of culture. To quantify colony-forming efficiency, the total number of organoids per well was counted manually 7 days after seeding using a light microscope at ×20 magnification. Organoid diameter was measured 14 days after seeding with a light microscope connected to NIS-Elements software (Nikon Europe, Netherlands). For immunofluorescence, organoid cultures were fixed with ice-cold acetone/methanol (1:1) for 12 min at −20°C, then non-specific binding sites were blocked in PBS with 5% bovine serum albumin (BSA; Sigma). Samples were stored at 4°C until immunofluorescence analysis.

### Immunofluorescence staining

Immunofluorescence staining was performed as described before (Ng-Blichfeldt et al., 2019; Wu et al., 2019). Cultures were incubated with primary antibodies (alveolar cells: anti-proSP-C Millipore AB3786; ciliated cells: anti-Acetylated tubulin Sigma T6793) diluted in PBS with 0.1% (wt/vol) BSA and 0.1% (vol/vol) Triton X-100 at 4°C overnight, then washed three times in PBS (> 20 min between washes), after which cultures were incubated with secondary antibodies and DAPI at 4°C overnight. After 3x washing with PBS, transwell membranes were excised from inserts and mounted on glass slides with mounting media and glass coverslips. Immunofluorescence was visualized using a Leica SP8 confocal microscope (Wetzlar, Germany), and images were obtained with Leica Application Suite software. Per condition, > 150 organoids were analyzed at ×40 magnification, and the number of alveolar vs airway organoids was quantified (Ng-Blichfeldt et al., 2019; Hu et al., 2020).

### TOP/FLASH reporter assay

The TOP/FOP flash assay was performed as described before (Baarsma et al., 2017; Wu et al., 2019). In short, MRC-5 fibroblasts were seeded in 96-well plates, and upon confluence, were transfected with 100 ng/well TOP luciferase reporter plasmid or negative control (FOP plasmid) using Lipofectamine™ LTX Reagent with PLUS™ Reagent (Invitrogen, Carlsbad, United States) in serum-free Opti-MEM^®^ medium (Life Technologies, Carlsbad, United States) for 5 h. Thereafter, MRC-5 cells were stimulated for 6 h with vehicle, 2 or 5 AU/mL freshly prepared CSE (15 min exposure washed and incubated), 100 nM Tg without or with 2 µM CHIR99021 and/or 10 mM NAC in Opti-MEM^®^ medium with 0.1% FBS. After incubation, MRC-5 fibroblasts were lysed using the Bright-Glo™ Luciferase Assay System (Promega) and luciferase activity was measured using a Spectramax Microplate Reader (Molecular Devices, San Jose, United States). SoftmaxPro (Molecular Devices) was used to collect and analyze data.

### q-PCR analysis

RNA was isolated from MRC-5 fibroblasts according to the manufacturer’s instruction using Maxwell RNA extraction kits (Promega, Madison, WI, United States). Quantitative RT-PCR (q-PCR) was performed as described previously (Delbrel et al., 2019). q-PCR reactions were performed in triplicate. We included two reference genes, selected using the “Genorm method” (Genorm, Primer design, Southampton, United Kingdom), to calculate the normalized gene expression. Expression values were determined by the relative gene expression of a standard curve as determined by CFX manager software, and expressed as fold increase (Bio-Rad). Used primer pairs are listed in Table 2.

**TABLE 2:** Primer pairs used for gene expression analysis.

Gene (human)	Forward primer sequence (5′ to 3′)	Reverse primer sequence (3′ to 5′)
*ATP5B*	TCA​CCC​AGG​CTG​GTT​CAG​A	AGT​GGC​CAG​GGT​AGG​CTG​AT
*RPL13A*	AAG​GTG​GTG​GTC​GTA​CGC​TGT​G	CGG​GAA​GGG​TTG​GTG​TTC​ATC​C
*AXIN2*	CGGGAGCCACACCCTTCT	TGG​ACA​CCT​GCC​AGT​TTC​TTT
*GADD34*	ATG​TAT​GGT​GAG​CGA​GAG​GC	GCA​GTG​TCC​TTA​TCA​GAA​GGC
*Bip*	CGA​GGA​GGA​GGA​CAA​GAA​GG	CAC​CTT​GAA​CGG​CAA​GAA​CT
*CHOP*	GCA​CCT​CCC​AGA​GCC​CTC​ACT​CTC​C	GTC​TAC​TCC​AAG​CCT​TCC​CCC​TGC​G
*XBP1SPL*	TGC​TGA​GTC​CGC​AGC​AGG​TG	GTC​GGC​AGG​CTC​TGG​GGA​AG
*HGF*	TCC​AGA​GGT​ACG​CTA​CGA​AGT​CT	CCC​ATT​GCA​GGT​CAT​GCA​T
*FGF2*	AGA​AGA​GCG​ACC​CTC​ACA​TCA	CGG​TTA​GCA​CAC​ACT​CCT​TTG
*FGF7*	TCC​TGC​CAA​CTT​TGC​TCT​ACA	CAG​GGC​TGG​AAC​AGT​TCA​CAT
*FGF10*	CAG​TAG​AAA​TCG​GAG​TTG​TTG​CC	TGA​GCC​ATA​GAG​TTT​CCC​CTT​C
*HMOX1*	AAG​ACT​GCG​TTC​CTG​CTC​AAC	AAA​GCC​CTA​CAG​CAA​CTG​TCG
*Wnt5A*	GGG TGG GAA CCA AGA AAA AT	TGG AAC CTA CCC ATC CCA TA
*WNT5B*	ACG CTG GAG ATC TCT GAG GA	CGA GGT TGA AGC TGA GTT CC

Gene (murine)	Forward primer sequence (5′ to 3′)	Reverse primer sequence (3′ to 5′)
*Xbp1spl*	CTG​AGT​CCG​CAG​CAG​GTG​CAG	GTC​CAT​GGG​AAG​ATG​TTC​TGG
*Chop*	GGA​GCT​GGA​AGC​CTG​GTA​TGA​G	GCA​GGG​TCA​AGA​GTA​GTG​AAG​G
*Gadd34*	CCC​GAG​ATT​CCT​CTA​AAA​GC	CCA​GAC​AGC​AAG​GAA​ATG​G
*Axin2*	CGC​TTT​GAT​AAG​GTC​CTG​GC	AGT​TCC​TCT​CAG​CAA​TCG​GC
*Hmox1*	GAT​AGA​GCG​CAA​CAA​GCA​GAA	CAG​TGA​GGC​CCA​TAC​CAG​AAG
*Actb*	GTG​ACG​TTG​ACA​TCC​GTA​AAG​A	GCC​GGA​CTC​ATC​GTA​CTC​C

### Statistical analyses

Data are presented as mean ± SEM. *N* refers to number of independent experiments using MRC-5 fibroblasts, and n refers to number of independent experiments upon EpCAM^+^ isolation. Data were analyzed using One-way or Two-way ANOVA (as indicated in the legends) and Tukey *post hoc* test to compare differences amongst groups. All analyses were performed using Graphpad Prism 9.3.1. Differences at a value of *p* < 0.05 were considered statistically significant.

## Results

### CSE induces cellular stress responses in MRC-5 lung fibroblasts, whilst impairing CHIR99021-induced Wnt/β-catenin signalling

**FIGURE 2:**
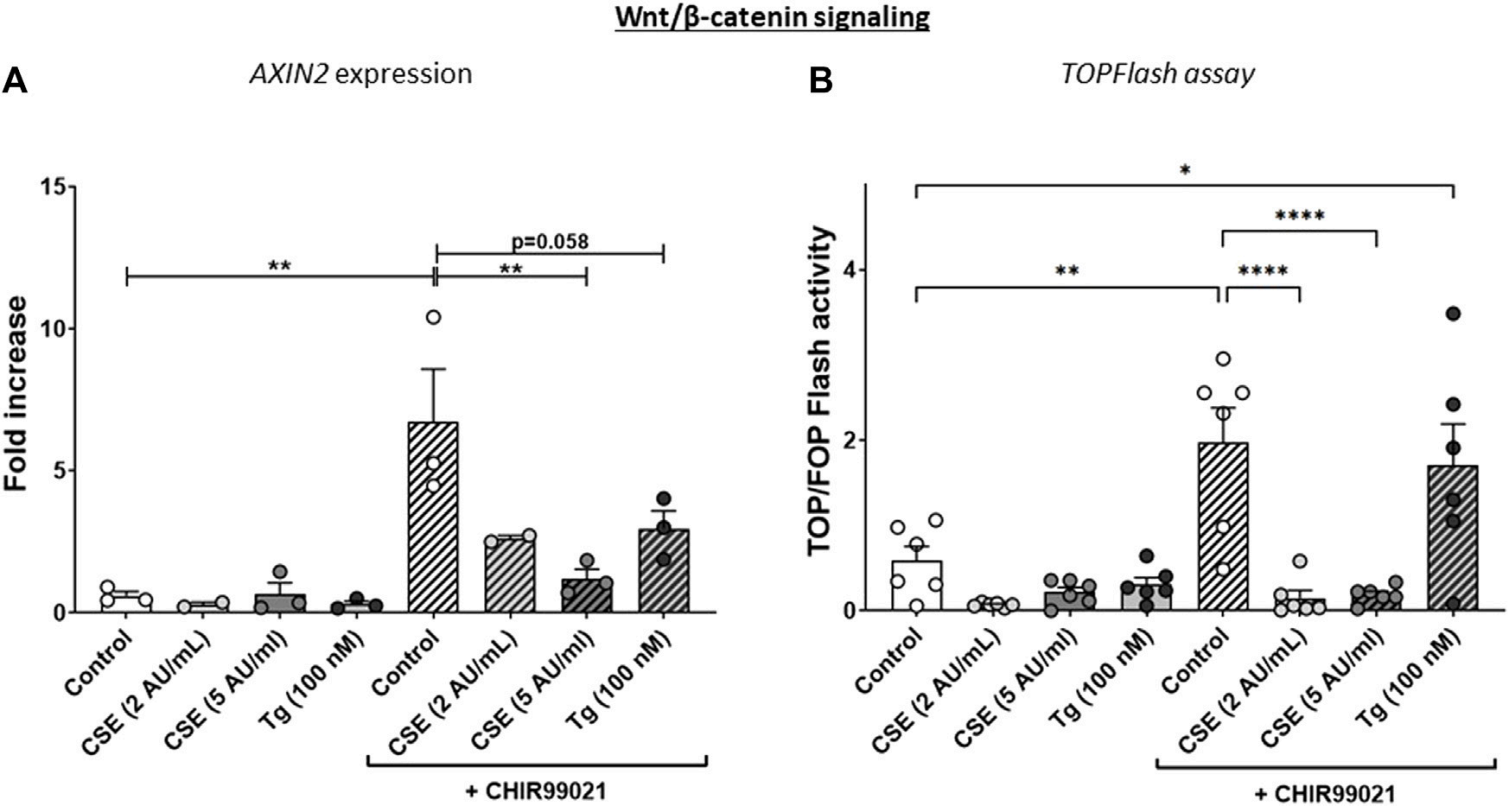
**CSE lowers CHIR99021-induced Wnt/b-catenin signalling in MRC-5 lung fibroblasts.** MRC-5 fibroblasts were cultured until confluency was reached, after which they were serum-starved for 24h. Upon starvation, MRC-5 fibroblasts were exposed to vehicle (white dots/bars) freshly-prepared CSE (2 or 5 AU/mL, light and dark grey dots/bars resp.) for 15 min or Tg (dark grey dots/grey bars) and stimulated for 6 h. CHIR99021 or vehicle (2 μM, stripes bars) was added during stimulation with CSE or Tg. Cells were then lysed for RNA isolation, cDNA synthesis and RT-qPCR. The effect on Wnt/β-catenin signalling was assessed by determining mRNA expression of AXIN2 **(A)** and using a TOP/FLASH reporter **(B)**. Data are shown as mean ± SEM; N = 3-4 independent experiments; *p < 0.05, **p < 0.01, ***p < 0.001, ****p < 0.0001. Data were analyzed using One-way ANOVA statistical testing in Graphpad Prism 9.3.1. Abbreviations: AU/mL: arbitrary units/mL; CSE: cigarette smoke extract; Tg: thapsigargin.

The experimental set-up is visualized in Figure 1A. MRC-5 lung fibroblast were exposed to 5 AU/mL CSE for 15 min and subsequently incubated for 6 h, and a CSE-induced expression of *HMOX1* (Figure 1B) was observed. Tg, a widely used nonspecific activator of the UPR, clearly increased expression of *GADD34*/*PPP1R15A*, *CHOP, XBP1spl* and *BiP* (Figures 1C, D, Supplementary Figure S1A), whereas CSE exposure only significantly increased *GADD34*/*PPP1R15A* expression (Figures 1C, D), and therefore we cannot conclude that CSE activates the UPR and/or ISR. As activation of the Wnt/β-catenin pathway is suggested to tip the balance towards a pro-repair and regenerative response in both epithelial cells and fibroblasts in COPD (Kneidinger et al., 2011; Hu et al., 2020), we treated MRC-5 lung fibroblasts with the Wnt/β-catenin activating compound, CHIR99021 (2 µM), during stimulation with CSE and Tg. Activation of the Wnt/β-catenin pathway did not modulate CSE-induced *GADD34*/*PPP1R15A* and *HMOX1* expression. However, CSE inhibited CHIR-induced activation of the Wnt/β-catenin target gene *AXIN2*, whereas the inhibitory effect of Tg on CHIR-induced *AXIN2* was less pronounced (Figure 2A). This inhibitory effect of CSE on CHIR99021-induced Wnt/β-catenin signalling activation was confirmed in a TOP/FLASH reporter assay (Figure 2B).

To determine whether the CSE-mediated reduction of CHIR99021-induced Wnt/β-activation was dependent on components of the UPR and/or ISR pathways, we used mouse embryonic fibroblasts (MEF) from wildtype, *Gadd34−/−*, *Chop−/−* or EIF*2a (AA)* signalling deficient mice (Supplementary Figure S1A). In line with our observations in human MRC-5 cells, *Gadd34* and *Hmox1* expression was increased upon CSE exposure in MEFs, whereas CSE did not increase *Xbp1spl* (data not shown). In contrast to MRC-5 fibroblasts, CSE did increase *Chop* expression in MEFs (data not shown). CHIR99021-induced activation of Wnt/β-catenin signalling appeared less pronounced in MEF. Importantly, absence of Gadd34 or Chop did not affect CSE- or Tg-induced impairment in CHIR-induced responses (Supplementary Figures S1B–E), whereas this impairment was less clear in MEFs unable to phosphorylate eIF2α. These data suggest that Gadd34 and CHOP may not be required for the suppressive effect of CSE on activation of CHIR99021-induced Wnt/β-catenin signalling, whereas oxidative stress may be involved in this effect.

### CSE exposure impairs repair-related factors

**FIGURE 3:**
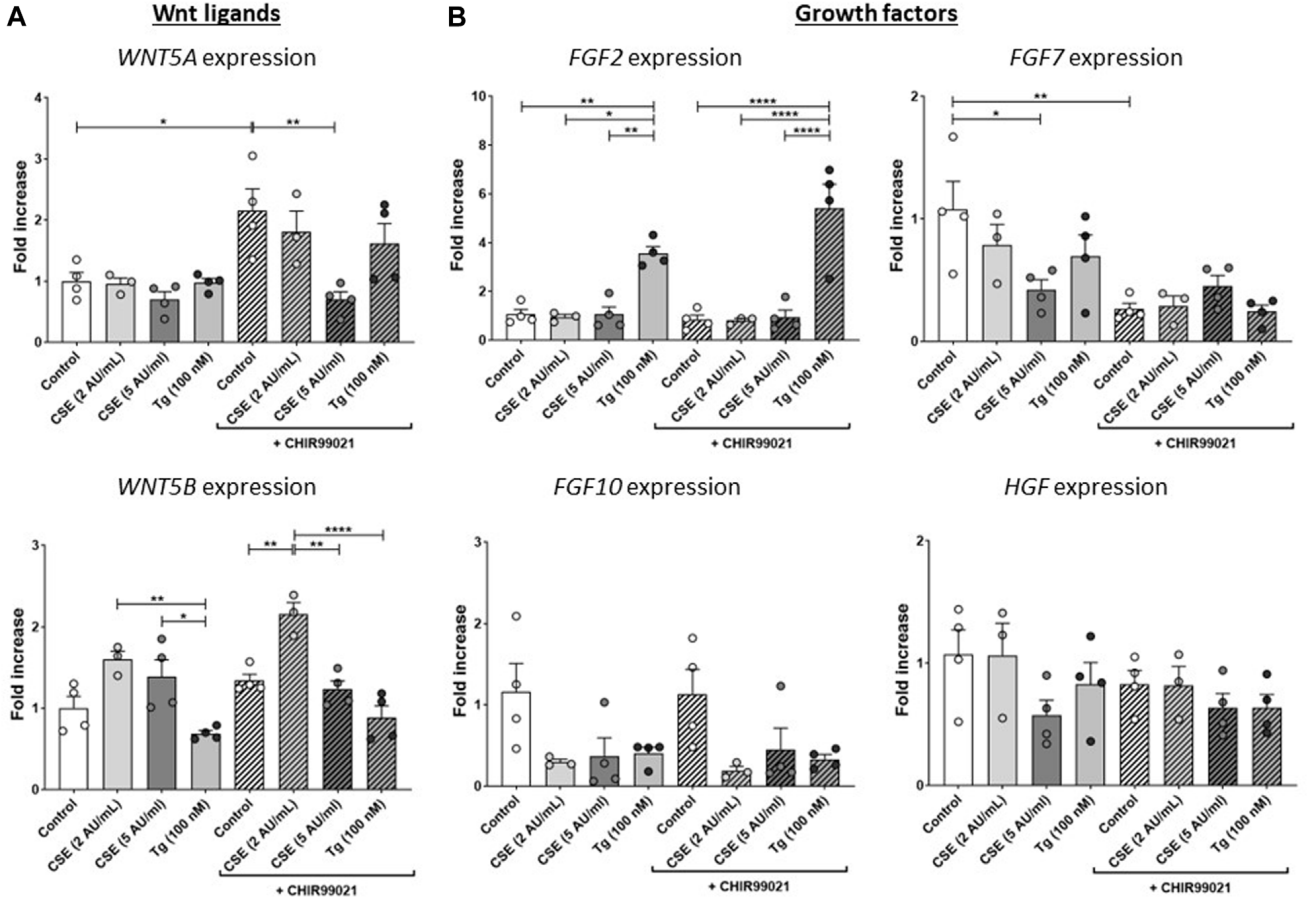
**CSE impairs repair-related factors.** MRC-5 fibroblasts were cultured until confluency was reached, after which they were serum-starved for 24 h. Upon starvation, MRC-5 fibroblasts were exposed to vehicle (white dots/bars) freshly-prepared CSE (2 or 5 AU/mL, light and dark grey dots/bars resp.) for 15 min or Tg (dark grey dots/grey bars) and stimulated for 6 h. Cells were then lysed for RNA isolation, cDNA synthesis and RT-qPCR. Expression of Wnt ligands (WNT5A and WNT5B) **(A)** and growth factors (FGF2, FGF7, FGF10 and HGF) **(B)** were determined as a read-out of repair-related factors. Data are shown as mean ± SEM; N = 3–4 independent experiments; *p < 0.05, **p < 0.01, ***p < 0.001, ****p < 0.0001. Data were analyzed using One-way ANOVA statistical testing in Graphpad Prism 9.3.1. Abbreviations: AU/mL: arbitrary units/mL; CSE: cigarette smoke extract; FGF: fibroblast growth factor; HFG: hepatocyte growth factor; Tg: thapsigargin.

To further understand the effects of CSE and Tg exposure on MRC-5 fibroblast and their suggested supportive function for epithelial cells, we measured expression of several growth factors and Wnt ligands related to (impaired) repair. Expression of *WNT5A* was induced upon treatment with CHIR99021 compared to control, and exposure to 5 AU/mL CSE, but not Tg, reduced CHIR99021-induced *WNT5A* expression (Figure 3A). *WNT5B* was increased significantly upon exposure to 2 AU/mL CSE and CHIR99021 treatment compared to control and exposure to 5 AU/mL and Tg. Interestingly, also CHIR99021 alone significantly reduced *FGF7* expression, which was not further reduced by CSE or Tg. These findings suggest that CSE and Tg both alter expression of repair-related mediators in MRC-5 cells, which could not all be restored by CHIR99021-induced Wnt/β-catenin activation.

### Exposure to CSE and ER stress in MRC-5 fibroblasts impairs support of epithelial organoid formation, which is partly restored by treatment with the Wnt/β-catenin activator CHIR99021

**FIGURE 4:**
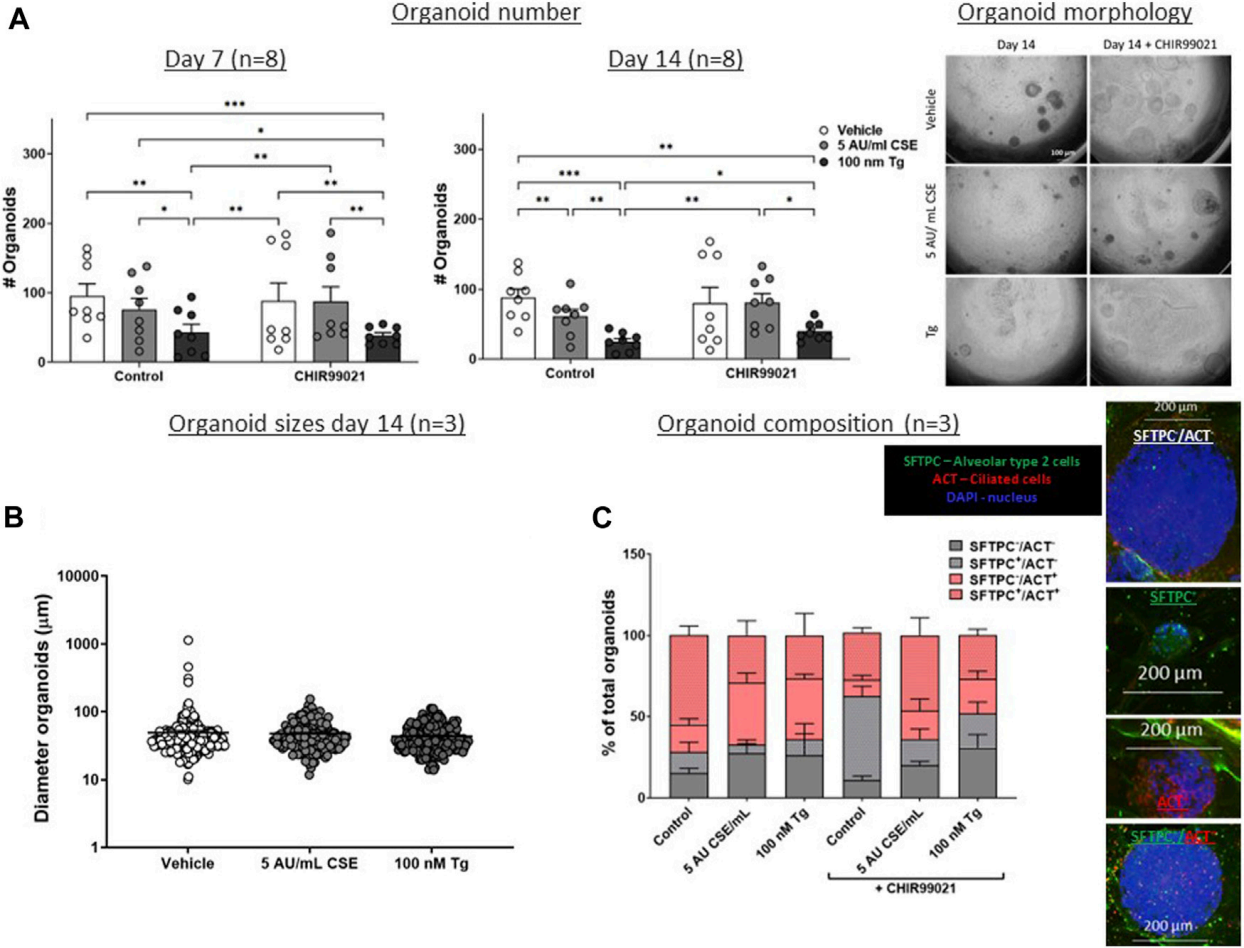
**CSE and Tg pre-exposure of MRC-5 fibroblasts impairs mesenchymal support of epithelial organoid formation.** MRC-5 fibroblasts were mitomycin-C inactivated for 2 h, followed by washing and a 1h recovery-period. Upon starvation, MRC-5 fibroblasts were exposed to vehicle (white dots/bars) freshly-prepared CSE (5 AU/mL, ldark grey dots/bars) for 15 min or Tg (dark grey dots/dark grey bars) and stimulated for 6 h. MRC-5 cells were then detached and seeded with freshly isolated mouse Epcam + lung epithelial cells in a 1:1 ratio in Matrigel, and organoid formation and growth was followed for 14 days. Organoid number (white arrows) was counted at day 7 and 14 **(A)**. Data are shown as mean ± SEM; N = 4 independent MRC-5 fibroblast experiments, EPCAM^+^ cells: *n* = 8 mice; *p < 0.05, **p < 0.01, ***p < 0.001. Organoid size was determined at day 14 **(B)** in, and organoid composition was determined using immunofluorescence staining of organoids fixed at day 14 organoids. Organoids were costained for proSFTPC (alveolar, green) and ACT (bronchial, red) and displayed as percentage of total organoids expressing one, both, or neither markers **(C)** (for both *n* = 3 mice). The nuclei were visualized using DAPI (blue). Data presented in 4B were analyzed using One-way ANOVA statistical testing in Graphpad Prism 9.3.1. Data presented in Figures 4A,C were analyzed using Two-way ANOVA statistical testing in Graphpad Prism 9.3.1. Abbreviations: ACT: acetylated tubulin; AU/mL: arbitrary units/mL; CSE: cigarette smoke extract; SFTPC: pro-surfactant protein-C; Tg: thapsigargin.

As Wnt/β-catenin signalling as well as expression of repair related factors was altered upon CSE/Tg exposure in MRC-5 fibroblasts, we performed a lung epithelial organoid assay in order to assess the functional consequence on lung epithelial progenitor function. Therefore, mitomycin-treated MRC-5 fibroblasts were stimulated with 5 AU/mL CSE or Tg to induce ER stress, and subsequently seeded in Matrigel with murine lung EpCAM^+^ epithelial cells (Figure 1A). Organoid formation was followed for 14 days. Pre-exposure of MRC-5 lung fibroblast to Tg resulted in a lower number of epithelial organoids both at day 7 and day 14, while CSE pre-exposure lowered epithelial organoid formation only at day 14 (Figure 4A); organoid size and composition was unaffected (Figures 4B, C). After showing that CSE and Tg impair CHIR-induced Wnt-signalling based on analysis of *AXIN2* expression (Figure 2), we next investigated whether residual Wnt signalling sufficed to increase organoid formation. To this end, MRC-5 lung fibroblasts were treated with 2 µM CHIR99021 during stimulation with CSE and Tg and effects on support of epithelial organoid formation were assessed. Remarkably, CHIR99021 pre-treatment of MRC-5 fibroblasts alone did not increase organoid numbers, but pre-treatment with CHIR99021 during CSE exposure restored support of epithelial organoid formation (Figure 4A), which was evident most clearly at 14 days. Also, CHIR99021-pre-treatment of MRC-5 cells appeared to induce a more mature organoid phenotype with higher numbers of SFTPC^+^ cells compared to CHIR99021-untreated organoids, although this effect was not significant.

These findings suggest that Tg and CSE pre-exposure of MRC-5 lung fibroblasts impairs their ability to support epithelial organoid formation. CSE-induced impairment of support of epithelial organoid formation and maturation of organoids was partly restored upon pre-treatment with the Wnt/β-catenin activator CHIR99021, suggesting that activation of Wnt/β-catenin signalling during CSE exposure reverses some of the CSE-induced anti-repair effects.

### Treatment with the oxidative stress inhibitor, N-acetylcysteine, partly restores effects of CSE exposure

**FIGURE 5:**
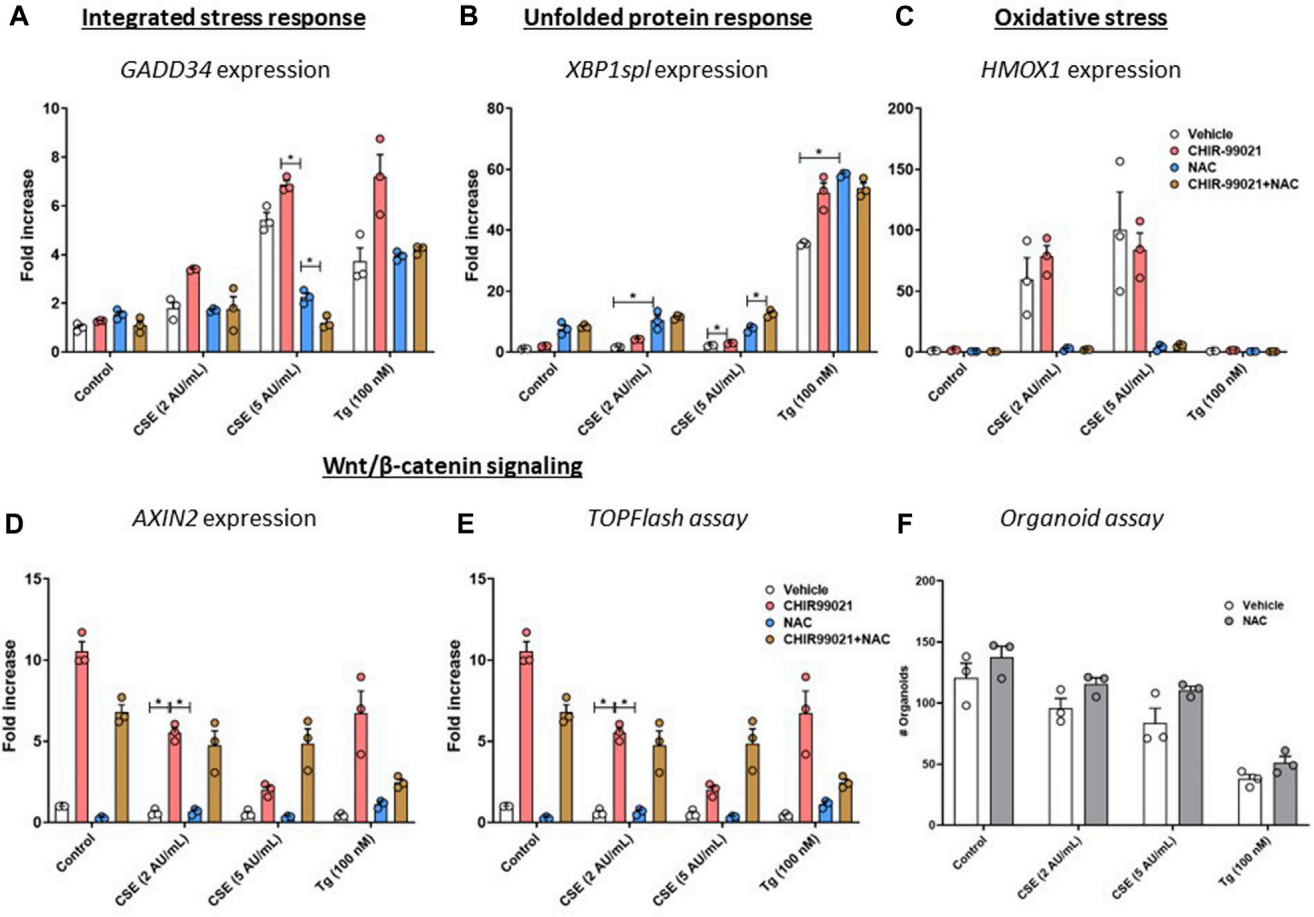
**Pre-treatment with an anti-oxidant does not restore impaired Wnt/β-catenin signalling upon CSE exposure.** MRC-5 fibroblasts were cultured until confluency was reached, after which they were serum-starved for 24h. Upon starvation, MRC-5 fibroblasts were exposed to freshly-prepared CSE (2 or 5 AU/mL) for 15 min or Tg and stimulated for 6h. CHIR-99021 (2 μM, pink dots and bars) or vehicle (white dots and bars) and (brown dots and bars)/or N-acetylcysteine (10 mM, blue dots and bars) was added during stimulation with CSE or Tg. Cells were then lysed for RNA isolation, cDNA synthesis and RT-qPCR. Gadd34 **(A)** mRNA expression were determined as a read-out for activation of the ISR, whereas XBP1spl **(B)** was measured to determine UPR activation. HMOX1 expression **(C)** was measured as marker for oxidative stress and the effect on Wnt/β-catenin signalling was assessed by determining mRNA expression of AXIN2 **(D)** and using a TOP/FLASH reporter **(E)**. MRC-5 fibroblasts were mitomycin-C inactivated for 2 h, followed by washing and a 1 h recovery-period. MRC-5 fibroblasts were subsequently exposed to freshly-prepared CSE (5 AU/mL) for 15 min, Tg or Tm and stimulated for 6 h. N-acetylcysteine (10 mM) or vehicle was added during stimulation with CSE, Tg or Tm. MRC-5 cells were then detached and seeded with freshly isolated mouse Epcam + lung epithelial cells in a 1:1 ratio in Matrigel, and organoid formation and growth was followed for 14 days; organoid number was counted at day 14 **(F)**. Data are shown as mean ± SEM; *n* = 3 mice; *p < 0.05. Data presented in **(A–F)** were analyzed using Two-way ANOVA statistical testing in Graphpad Prism 9.3.1. Abbreviations: AU/mL: arbitrary units/mL; CSE: cigarette smoke extract; NAC: N-acetylcystein; Tg: thapsigargin.

To determine the role of oxidative stress in the observed effects on Wnt/β-catenin signalling activation and support of organoid formation, MRC-5 fibroblasts were treated with CHIR99021 (2 µM) and/or N-acetylcysteine (NAC, 10 mM) or vehicle during the 6 h stimulation with CSE (2 or 5 AU/mL, 15 min exposure) or 100 nM Tg. There were no differences in expression of *GADD34*/*PPP1R15A* upon NAC or NAC + CHIR99021 pre-treatment (Figure 5A) compared to vehicle control. In contrast, *XBP1spl* was upregulated upon NAC pre-treatment of CSE-exposed cells (Figure 5B). Although non-significant, NAC pre-treatment reduced CSE-induced expression of *HMOX1* (Figure 5C). mRNA expression of *AXIN2* (Figure 5D) was unaltered upon NAC pre-treatment, which was confirmed using a Wnt/β-catenin reporter (Figure 5E). Despite our observation that NAC prevented CSE-induced effects on *GADD34*/*PPP1R15A*, and *HMOX1*, pre-treatment of MRC-5 cells with NAC did not affect epithelial organoid formation (Figure 5F). These findings suggest that the observed CSE-mediated inhibition of support of epithelial organoid formation (Figure 2A) only partly results from oxidative stress.

## Discussion

Crosstalk between fibroblasts and lung epithelial cells is crucial for both lung development and appropriate repair responses following lung injury, and this beneficial interaction has been suggested to be impaired in chronic lung diseases such as COPD. In this study, we show that exposure to cigarette smoke extract (CSE) of MRC-5 lung fibroblasts induced cellular stress responses, including oxidative stress and upregulation of *GADD34*/*PPP1R15A*. Furthermore, we showed that CSE caused an impairment of the ability of MRC-5 cells to support of epithelial progenitor function, as assessed by the organoid forming capacity of lung EpCAM^+^ cells. This effect was partially restored upon activation of the Wnt/β-catenin signalling pathway in fibroblasts during CSE stimulation.

Previous studies showed that CSE exposure induces myofibroblast differentiation of MRC-5 fibroblasts as assessed by enhanced α-SMA expression (Baek et al., 2012; Song et al., 2019) and altered expression profiles of proteins related to stress responses, mitochondrial activity, and aging in human fetal lung fibroblasts (HFL-1) (D'Anna et al., 2015). Myofibroblast activation has been associated with impaired repair and remodelling, and may contribute to progression of COPD and ILD (Ng-Blichfeldt et al., 2019; Liu et al., 2021). We showed that exposure to CSE induced expression of *GADD34*/*PPP1R15A* in MRC-5 lung fibroblasts, whereas UPR markers were unaffected. These findings are in contrast to previously reported findings using MRC-5 fibroblasts, in which the UPR was found to be activated upon CSE stimulation (Song et al., 2019). This apparent discrepancy may result from differences in CSE preparation method, CSE dosage and CSE exposure duration. Exposure to the chemical ER stress inducer Tg did activate markers of both the ISR and the UPR, which is in line with other studies. However, it needs to be noted that Tg may also activate other cellular processes unrelated to the UPR and ISR by its effect on intracellular calcium stores.

We showed that CSE and TG not only induced cellular stress responses in MRC-5 cells, but also decreased the capacity of the MRC-5 fibroblasts to support epithelial organoid formation. Furthermore, we showed that pre-exposure of MRC-5 cells to CSE resulted in (non-significantly) diminished expression of maturation markers in the organoids, as shown by an increase in SFTPC^−^/ACT^−^ organoids. Interestingly, CHIR99021 treatment was able to partially restore this inhibitory effect of CSE treatment, although CSE pre-treatment did lower ((but did not abolish) CHIR-99021-induced *AXIN2* expression as marker of Wnt/β-catenin signalling activation compared to control.

The reduced ability of MRC-5 fibroblasts to support organoid formation upon CSE or Tg exposure may result from a cascade of events, including impaired growth factor production and activation of cellular stress signals. TGF-β is increased in COPD lungs and contributes to myofibroblast formation (Konigshoff et al., 2009; Baarsma et al., 2011). Furthermore, we previously showed that fibroblast exposure to TGF-β decreases their capacity to support epithelial organoid formation (Ng-Blichfeldt et al., 2019), which results from altered expression of Wnt/β-catenin signalling components and target genes. Our results using Tg indicate that ER stress reduced CHIR99021-induced Wnt/β-catenin signalling activation. Whereas acute ER stress is important in maintaining cellular homeostasis (Marciniak, 2019), prolonged ER stress responses may result in cellular dysfunction and cell death and is closely linked to other cellular stress responses including oxidative stress, mitochondrial dysfunction and cellular senescence (Kanaji et al., 2014; Koloko Ngassie et al., 2021), processes that are increased in lungs from COPD patients. Fibroblasts from COPD patients show disorganized organelle organization and enhanced ER stress (Weidner et al., 2018), which may impact the niche-function of fibroblasts in lung epithelial repair. ER stress may interfere with Wnt/β-catenin signalling in various ways. Firstly, ER stress may interfere with Wnt ligand processing and secretion in Wnt-producing cells, leading to lower production or inactivity of secreted Wnt ligands (Verras et al., 2008). Secondly, ER stress leads to attenuation of protein translation resulting in a transient decrease in short-lived signalling molecules including bone morphogenic protein-4 (BMP-4) (Malzer et al., 2018) and potentially β-catenin. ER stress may also inhibit transcription of proteins downstream of β-catenin (van Lidth de Jeude et al., 2017), and finally ER stress responses may lead to proteosomal degradration of proteins (Fabre et al., 2019). There are thus several mechanisms by which ER stress may interfere in the Wnt/β-catenin signalling pathway, although the precise mechanism is unclear yet.

Although we did not find altered gene expression of β-catenin upon CSE- or Tg-induced ER stress (not shown), we did show that gene expression of various injury- and repair-related factors (*FGF7*, *WNT5A*) and CHIR99021-induced Wnt/β-catenin signalling activation was altered upon CSE exposure in MRC-5 cells. This is in line with previous findings showing that the mesenchymal niche secretes fibroblast growth factors (FGFs), Wnt ligands/signalling activation and other growth factors (Frank et al., 2016; Zepp et al., 2017; Khedoe et al., 2021; Penkala et al., 2021), and thereby orchestrates alveolar cell function. We did not measure protein levels of secreted growth factors, due to the short-lived nature of especially Wnt ligands, but do show a functional impairment in support of epithelial organoid formation. Various FGFs have been shown to enhance lung epithelial organoid formation (Rabata et al., 2020), and HGF and FGF7 were able to reverse diminished organoid growth upon co-culture with TGF-β-treated MRC-5 fibroblasts (Ng-Blichfeldt et al., 2019). Future studies are needed to define whether altered expression of these growth factors contributes to impaired repair in COPD. Furthermore, additional factors induced by CSE-exposure in MRC-5 cells, including pro-inflammatory factors, may have contributed to the impaired capacity to support lung epithelial progenitor function, which we did not study here.

Cigarette smoke induces oxidative stress and various studies suggest that oxidative stress may alter mitochondrial and ER function (Fabre et al., 2019) and Wnt/β-catenin signalling (Qu et al., 2019). Therefore, we investigated whether oxidative stress mediates the CSE-induced reduction in CHIR99021-induced *AXIN2* expression. Reducing oxidative stress by NAC treatment has been shown to restore diesel-induced impaired lung epithelial organoid formation (Wu et al., 2022). Whereas we show that NAC treatment did reduce the oxidative stress (e.g., *HMOX1* mRNA expression), NAC only partly restored *AXIN2* expression and did not improve organoid supporting capacity of CSE-exposed MRC-5 fibroblasts, suggesting that the observed effects may be (partly) independent from oxidative stress. It has to be noted, that NAC not only has anti-oxidant activities, but also breaks disulfide bridges (Aldini et al., 2018). Previous research showed that DTT, which also breaks disulfide bridges, induces ER stress (Sundaram et al., 2018), which is in line with our observation that NAC increased *XBP1spl* in CSE- and Tg-exposed cells. Furthermore, oxidative stress has been shown to alter Wnt/β-catenin signalling (directly) by various mechanisms, which is discussed in detail elsewhere (Qu et al., 2019), and the direct interaction between oxidative stress and Wnt/β-catenin signalling activation is beyond the scope of the current study. CSE exposure induces various cellular stress responses, and CHIR99021-induced Wnt/β-catenin signalling activation was lowered by CSE exposure. However the relative contribution of oxidative stress, ER stress and factors related to repair to the ability of CSE to impair WNT/β-catenin signalling and organoid growth is unclear.

Whereas we show a clear effect of CSE and ER stress on mesenchymal cellular responses and their ability to support of epithelial organoid formation, there are several limitations of our model. We used a single exposure of CSE in MRC-5 cells. Future studies using repeated exposures may better mimic COPD pathogenesis. Here, we used MEF to determine the role of CSE-mediated reduction of CHIR-induced Wnt/β-catenin activation, and showed that MEFs deficient in components of the UPR or ISR, did not respond differently to CSE exposure compared to WT MEFs. A limitation could be that we did not use lung fibroblasts from mice deficient in UPR/ISR components. However, as WT MEF exhibited similar responses as MRC-5 fibroblasts, we do not expect large differences in responses. Furthermore, mice deficient in some of the UPR/ISR components present a lethal phenotype, and therefore experiments with lung fibroblasts for those mice was not feasible. Furthermore, heightened ER stress has also been shown in lung epithelial cells (Malhotra et al., 2009; Wang et al., 2017) and may also affect lung epithelial progenitor function (Goldfarbmuren et al., 2020). Here, we specifically investigated the cross-talk between mesenchymal cells and epithelial cells, as ER stress is also increased in fibroblasts of COPD patients (Weidner et al., 2018). Finally, we used Tg as a well-established activator of the UPR, that causes ER stress by depletion of Ca^2+^ stores. We cannot formally exclude the possibility that activities of Tg independent of UPR activation have contributed to our observations.

Despite these limitations, the findings from our study point to potential targets for improving altered cross-talk and lung epithelial repair in COPD. Studies have shown that CS-induced effects on ER stress and the subsequent activation of the UPR may be targeted through treatment with Tauroursodeoxycholic acid (TUDCA) (Tong et al., 2021) or 4-phenylbutyric acid (4-PBA) (Wang et al., 2017); treatment with these compounds prevented EMT in experimental fibrosis and experimental emphysema in mice. There are several other compounds available that also target the ER stress pathway, that may be studied in the context of lung epithelial repair. Our results show that the responses to ER stress and the ISR may partly mediate cigarette smoke-induced alterations in the cross-talk between mesenchymal cells and lung epithelial cells. Targeting ER stress or the ISR in fibroblasts may therefore represent an attractive therapeutic target to restore altered cross-talk and lung epithelial repair in COPD.

In conclusion, our study showed that CSE and ER stress impair Wnt/β-catenin activation in MRC-5 fibroblasts and subsequent cross-talk between lung fibroblasts and lung epithelial cells, resulting in impaired lung organoid formation at day 14. Thereby, we provide further insight into the way disturbed mesenchymal cell function in COPD may contribute to the impairment of repair processes observed in COPD.

## Data Availability

The original contributions presented in the study are included in the article/Supplementary Material, further inquiries can be directed to the corresponding author.

## Abbreviations

ACT, acetylated tubulin; AU/mL, arbitrary units/mL; BMP-4, bone morphogenic protein-4; COPD, chronic obstructive pulmonary disease; CS, cigarette smoke; CSE, CS extract; eIF2-a, eukaryotic initiation factor-a; EMT, epithelial-to-mesenchymal transition; ER, endoplasmic reticulum; FGF, fibroblast growth factors; HFL-1, human fetal lung fibroblasts; HGF, hepatocyte growth factor; ILD, interstitial lung diseases; ISR, integrated stress response; MACS, Magnetic-activated cell sorting; MEF, mouse embryonic fibroblasts; NAC, N-acetylcysteine; NEAAS, non-essential amino acids; SFTPC, pro-surfactant protein-C; Tg, Thapsigargin; UPR, unfolded protein response.
