# Corrigendum: Cigarette smoke restricts the ability of mesenchymal cells to support lung epithelial organoid formation

**DOI:** 10.3389/fcell.2024.1452028

**Published:** 2024-07-19

**Authors:** P. P. S. J. Khedoe, W. A. A. M. van Schadewijk, M. Schwiening, J. P. Ng-Blichfeldt, S. J. Marciniak, J. Stolk, R. Gosens, P. S. Hiemstra

**Affiliations:** ^1^ Department of Pulmonology, Leiden University Medical Centre, Leiden, Netherlands; ^2^ Department of Medicine, Cambridge Institute for Medical Research, University of Cambridge, Cambridge, United Kingdom; ^3^ Department of Molecular Pharmacology, Groningen Research Institute of Pharmacy, University of Groningen, Groningen, Netherlands; ^4^ MRC Laboratory of Molecular Biology, Cambridge Biomedical Campus, Cambridge, United Kingdom

**Keywords:** cigarette smoke extract, lung epithelial organoid, COPD, fibroblasts, oxidative stress, ER stress

In the published article, an **Author** name was incorrectly written as J. P. Ng-Blichtfeldt. The correct spelling is “J. P. Ng-Blichfeldt”.

The authors apologize for this error and state that this does not change the scientific conclusions of the article in any way. The original article has been updated.

