# Supplementary material for: Cigarette smoke restricts the ability of mesenchymal cells to support lung epithelial organoid formation
Source: Front Cell Dev Biol. 2023 Sep 19;11:1165581. doi: 10.3389/fcell.2023.1165581 (PMC10546195; doi:10.3389/fcell.2023.1165581)
Supplement: Supplementary file 1 [file Image1.pdf]

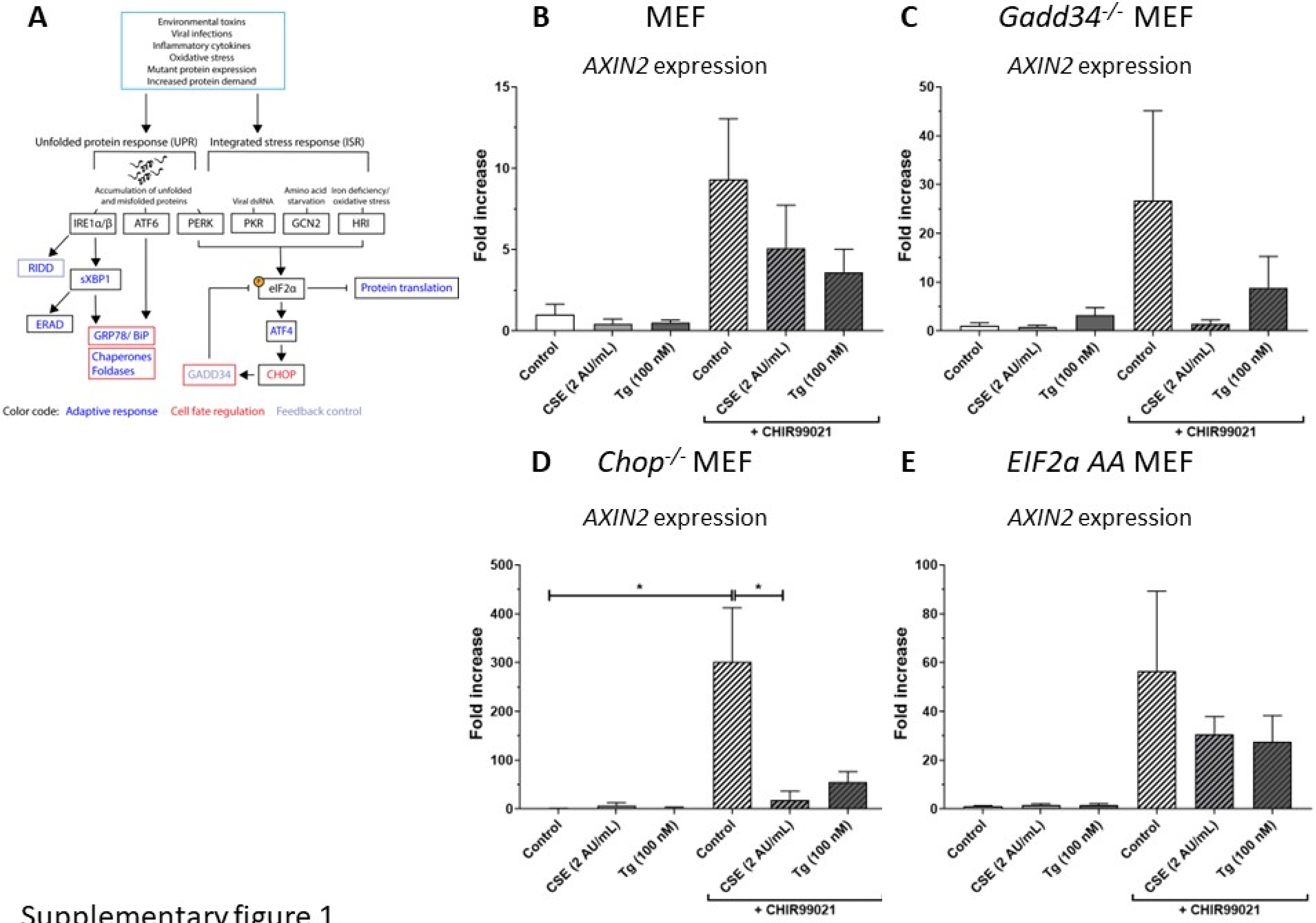

Supplementary figure 1

Supplementary Material

Supplemental figure 1: Absence of ER stress-related components does not restore impaired CHIR-induced Wnt/β-catenin signaling activation upon CSE exposure

To determine the role of ER stress-related components (A) in CSE-induced impaired Wnt/β-catenin signaling activation, mouse embryonic fibroblasts (MEF) from WT (B), Gadd34$^{-/-}$ (C), Chop$^{-/-}$ (D) or EIF2a AA signaling deficient (E) mice were cultured until confluency, after which they were serum-starved for 24h. Upon starvation, MEFs were exposed to freshly-prepared CSE (2 AU/mL) for 15 min or Tg and stimulated for 6h. CHIR99021 or vehicle (2 µM) was added during stimulation with CSE or Tg. Cells were then lysed for RNA isolation, cDNA synthesis and RT-qPCR, after which ER stress components (not shown) and AXIN2 expression were determined in the MEFs.
Data are shown as mean ± SEM; N= 3 independent experiments; *$p<0.05$
All data presented were analyzed using Two-way ANOVA statistical testing in Graphpad Prism 9.3.1.
Abbreviations: AU/mL: arbitrary units/mL; CSE: cigarette smoke extract; MEF: mouse embryonic fibroblasts; Tg: thapsigargin
